# Association of *STAT*-3 rs1053004 and *VDR* rs11574077 With FOLFIRI-Related Gastrointestinal Toxicity in Metastatic Colorectal Cancer Patients

**DOI:** 10.3389/fphar.2018.00367

**Published:** 2018-04-13

**Authors:** Elena De Mattia, Erika Cecchin, Marcella Montico, Adrien Labriet, Chantal Guillemette, Eva Dreussi, Rossana Roncato, Alessia Bignucolo, Angela Buonadonna, Mario D’Andrea, Luigi Coppola, Sara Lonardi, Eric Lévesque, Derek Jonker, Félix Couture, Giuseppe Toffoli

**Affiliations:** ^1^Clinical and Experimental Pharmacology, Centro di Riferimento Oncologico, National Cancer Institute, Aviano, Italy; ^2^Pharmacogenomics Laboratory, Centre Hospitalier Universitaire de Québec, Research Center and Faculty of Pharmacy, Laval University, Québec, QC, Canada; ^3^Medical Oncology Unit, Centro di Riferimento Oncologico, National Cancer Institute, Aviano, Italy; ^4^Medical Oncology Unit, San Filippo Neri Hospital, Rome, Italy; ^5^Pathology Unit, San Filippo Neri Hospital, Rome, Italy; ^6^Medical Oncology Unit 1, Istituto Oncologico Veneto, Istituto Di Ricovero e Cura a Carattere Scientifico, Padua, Italy; ^7^Centre Hospitalier Universitaire de Québec Research Center, Faculty of Medicine, Laval University, Québec, QC, Canada; ^8^Division of Medical Oncology, Department of Medicine, Ottawa Hospital, University of Ottawa, Ottawa, ON, Canada

**Keywords:** STAT-3, VDR, genetic markers, irinotecan (CPT-11), gastrointestinal toxicity, colorectal cancer, inflammation, FOLFIRI

## Abstract

Pharmacogenomics has largely been applied to the personalization of irinotecan-based treatment, focusing mainly on the study of genetic variants in adsorption, distribution, metabolism, and excretion (ADME) genes. The transcriptional control of ADME gene expression is mediated by a set of nuclear factors responding to cancer-related inflammation, which could have pharmacological implications. The aim of the present study was to uncover novel genetic predictors of neutropenia and gastrointestinal toxicity risk among 246 haplotype-tagging polymorphisms in 22 genes encoding inflammation-related cytokines and transcriptional regulators of ADME genes. The study comprised overall more than 400 metastatic colorectal cancer (mCRC) patients treated with first-line FOLFIRI, grouped in a discovery and a replication cohorts. A concordant protective effect of *STAT-3* rs1053004 polymorphism against the risk of grade 3–4 gastrointestinal toxicity was observed in both the cohorts of patients (OR = 0.51, *p* = 0.045, *q* = 0.521 and OR = 0.39, *p* = 0.043, respectively). *VDR* rs11574077 polymorphism was demonstrated to affect both irinotecan biliary index (BI) and glucuronidation ratio (GR) by a pharmacokinetic analysis. This effect was consistent with an increased risk of grade 3–4 gastrointestinal toxicity in the discovery cohort (OR = 4.46, *p* = 0.010, *q* = 0.305). The association was not significant in the replication cohort (OR = 1.44, *p* = 0.601). These findings suggest an effect of *STAT-3* and *VDR* polymorphisms on FOLFIRI-related gastrointestinal toxicity. If prospectively validated as predictive markers, they could be used to improve the clinical management of mCRC.

## Introduction

Irinotecan in association with FOLFIRI regimen represents the standard of care for first-line treatment of mCRC (Fujita et al., 2015). Despite the established antineoplastic effectiveness of the FOLFIRI regimen, the sporadic occurrence of severe and occasionally life-threatening complications often causes a failure of the chemotherapy, negatively impacting patient care (Rothenberg et al., 2001). Exposure to active irinotecan metabolite SN-38 is the major cause of toxicity, with severe neutropenia and delayed diarrhea as the dose-limiting toxicity and great inter-individual variability (Gupta et al., 1994, 1997). In the last few years, pharmacogenomics has largely been applied to the personalization of CRC treatment, specifically focusing on the genetic variability in ADME genes (De Mattia et al., 2015) demonstrating the role of genetic markers in *UGT1A* and *ABC*, and *SLC* transporters, in combination with clinico-demographic features, in predicting FOLFIRI toxicity (Toffoli et al., 2006; Cecchin et al., 2009; Toffoli et al., 2010; De Mattia et al., 2013a; Levesque et al., 2013; Chen et al., 2015a,b). As in the case of *UGT1A1*^∗^28, these studies led to specific pharmacogenetic guidelines (Swen et al., 2011; Caudle et al., 2013). Nevertheless, the optimization of irinotecan-based therapy remains sub-optimal and under-explored targets may significantly contribute to determining the likelihood of severe complications after chemotherapy.

The expression of irinotecan-related ADME genes is controlled upstream by crucial transcription factors that respond to endobiotic and xenobiotic stimuli (e.g., inflammation response, drug administration) with a demonstrated effect on irinotecan bioavailability. In particular, the inflammatory state, a condition linked to some cancers, including CRC (Markman and Shiao, 2015), has been shown to affect the pharmacokinetic and pharmacodynamic parameters of various chemotherapeutics by modulating drug metabolic enzymes and ABC/SLC transporters (Cressman et al., 2012; Harvey and Morgan, 2014). This effect is mediated by transcriptional regulators, such as STAT-3 and NFκB1, whose activity is controlled by pro-inflammatory cytokine-induced signaling pathways (Ho and Piquette-Miller, 2006; Reuter et al., 2010). More recently, nuclear receptors (NRs), another class of transcriptional regulators (De Mattia et al., 2013b, 2016; Cecchin et al., 2016), have emerged as crucial regulators of ADME genes in the presence of cytokines released during the inflammation process (Chen et al., 2012; Cressman et al., 2012; De Mattia et al., 2013b). Altered transcriptional functionality could impact irinotecan drug pharmacokinetic and pharmacodynamic profiles via the regulation of ADME gene expression. In this context a significant effect on irinotecan exposure and clinical outcome of some genetic variants in *HNF1A* gene was previously reported (Labriet et al., 2017).

The present study addressed the effect of polymorphisms in genes encoding transcriptional regulators and pro-inflammatory cytokines impacting irinotecan-related ADME genes on the risk of toxicity. The aim of the study, adopting a TagSNP approach, was to evaluate the systemic variability of 22 transcriptional regulators and pro-inflammatory cytokines impacting irinotecan-related ADME genes to define novel genetic markers that may improve the prediction of the differential probability of developing neutropenia and gastrointestinal (GI) toxicities after FOLFIRI treatment. A discovery/replication study design was used with data on more than 400 mCRC patients treated with first-line FOLFIRI (Toffoli et al., 2006; Levesque et al., 2013).

## Materials and Methods

### Patient Cohorts and Treatment

The discovery cohort included prospectively enrolled Northeastern Italian mCRC patients undergoing first-line FOLFIRI treatment and homogenously followed up between February 2002 and November 2005 (Toffoli et al., 2006; Cecchin et al., 2009). Of the 267 subjects enrolled, 250 were found to be eligible and included in the final analysis of toxicity (Toffoli et al., 2006); DNA, clinical, and pharmacokinetic data were collected and described previously (Toffoli et al., 2006; Cecchin et al., 2009). Patients were treated with either the Tournigand-modified FOLFIRI regimen (Tournigand et al., 2004) (>90% of total) or the FOLFIRI regimen, both based on a 180 mg/m^2^ intravenous dose of irinotecan. Details were published previously on eligibility criteria and treatment modalities, as well as the procedures for evaluating toxicity and data collection (Toffoli et al., 2006). In the present study, toxicity end-points were grade 3–4 neutropenia and GI (diarrhea, nausea, or vomiting) toxicities, which represent the major irinotecan-related side effects. The worst event recorded during the entire course of chemotherapy was considered. Criteria for therapy delay/discontinuation were reported previously (Toffoli et al., 2006).

The replication cohort included 167 Eastern Canadian mCRC patients receiving FOLFIRI-based regimens. All patients received a 180 mg/m^2^ intravenous dose of irinotecan every 2 weeks, and 75 patients also received co-treatments: bevacizumab, an experimental drug, or a placebo. Details on eligibility, treatment modalities, and toxicity data collection were previously documented elsewhere (Levesque et al., 2013; Chen et al., 2015a,b).

In both cohorts, the severity of neutropenia and GI toxicities were evaluated prospectively and according to the National Cancer Institute Common Terminology Criteria for Adverse Events, version 3.0, criteria.

All the patients in the study were self-reported Caucasian. The study protocol complied with the ethical guidelines of the 1975 Declaration of Helsinki. The protocol was approved by the Comitato Etico Indipendente-Centro di Riferimento Oncologico di Aviano and the CHU de Quebec Ethics Committees. All patients provided written informed consent for the genetic analysis before entering the study. All experiments were carried out in accordance with the relevant guidelines and regulations of the CHU de Québec and Centro di Riferimento Oncologico di Aviano.

### Candidate Genes and Polymorphism Selection

Target genes were selected initially on the basis of a literature search (PubMed-MEDLINE) prioritizing transcriptional controllers and cytokines clearly implicated in the regulation of transporters and phase I and II enzymes during inflammation. Particular attention was paid to the modulation of membrane carriers (i.e., ABCB1, ABCC1, ABCC2, ABCG2, and SLCO1B1) and metabolic proteins (i.e., UGT1A, CES, and CYPs) strictly involved in the FOLFIRI drug pathway. Genetic variants for each candidate gene were chosen successively using the TagSNP approach and the genotype frequency data downloaded from the HapMap website^1^; the filter parameters were HapMap CEU database (release #27) and MAF ≥ 0.05. This search permitted to obtain records about variants located in the exonic and intronic regions of the genes. The Tagger program implemented in Haploview^2^ (Broad Institute, Cambridge, MA, United States) was then employed to predict the TagSNPs (*r*^2^ = 0.80). For each block of linkage polymorphisms, a TagSNP was picked. If the initially selected TagSNPs were not amenable to genotype assay development (see section “Genetic analysis” below), an alternative TagSNP that captured the same information was chosen. Polymorphisms that tagged only themselves and did not have a substitute tag were rejected. Finally, the panel of selected TagSNPs was integrated with additional variants located in the 5′- and 3′ untranslated region of the gene chosen by screening the NCBI dbSNP database^3^ (Genomic Build: hg19/GRCh37 [Feb 2009]) using the following criteria: PubMed citation and MAF ≥ 0.05 in HapMap CEU population. At the end of this bioinformatics workflow, a set of 246 molecular markers in 22 candidate genes encoding NRs (PXR, LXR-A/B, FXR, RXR-A/B/G, CAR, VDR, PPAR-A/G/D, HNF4A, and HNF1A), transcription factors and related pathways (STAT-3, NF-kB1, IKBKB, and CHUK), and key pro-inflammatory cytokines (TNF, IL-1B, IL-6, and IFNγ), were selected (Supplementary Table S1) and introduced into the pharmacogenetic analysis.

### Genetic Analysis

Genomic DNA was extracted from peripheral blood using the High Pure PCR Template Preparation Kit (Roche Diagnostics GmbH, Mannheim, Germany). DNA samples were genotyped using the Illumina BeadXpress platform based on Golden Gate chemistry and the allelic discrimination method based on the TaqMan system. A 192-plex and 48-plex Illumina VeraCode GoldenGate Genotyping Assay (Illumina, Inc., San Diego, CA, United States) was developed using the Assay Design Tool (ADT) available through Technical Support on the Illumina website^4^. Only the assays with a high final score (≥0.6) and optimal designability (=1) were considered compatible with successful GoldenGate genotyping and introduced into the final custom panel. Samples were prepared for the analysis according to the manufacturer’s protocol. VeraScan software (version 2.0) was employed for fluorescence detection and the GenomeStudio V2011.1 tool (Illumina, Inc.) for genotype clustering with a polymorphism call-threshold of 0.25 (on a scale of 0–1). Sample replicates were introduced into each analysis to assess the robustness of the output records and to provide duplicate data to aid in the redefinition of clustering. Only the DNA samples and polymorphisms with a call rate >90% were retained in the final report. The excluded markers and six residual polymorphisms of the selected pool were tested in an allelic discrimination reaction using predesigned TaqMan SNP genotyping assays. All commercial TaqMan assays were purchased from Applied Biosystems^5^ and the analyses performed using the Applera TaqMan Universal Master Mix on an ABI 7500 (AB Applied Biosystems, Foster City, CA, United States) according to the manufacturer’s instructions. Positive and negative control samples were included in each analysis. More details about the analytical procedures are available upon request.

Polymorphisms to be tested in the replication cohort were genotyped by the Canadian group using iPLEX matrix-assisted laser desorption/ionization time-of-flight mass spectrometry (Sequenom, San Diego, CA, United States). Negative controls and a 5% random sample duplicate population were used to ensure the robustness and reproducibility of the assay. All extension primers and PCR assays were designed using SpectroDESIGNER software (Sequenom, San Diego, CA, United States). Markers that could not be sequenced due to poor primer design or because they were located in duplicated regions were replaced with TagSNPs in complete linkage disequilibrium (LD) (*r*^2^ = 1.00).

### Pharmacokinetic Analysis

Pharmacokinetic data were available for 71 patients in the discovery cohort (Toffoli et al., 2006). The pharmacokinetic parameters of irinotecan, SN-38 (active form of irinotecan), SN-38G (inactive glucuronidation form of irinotecan), the GR, and BI were determined as reported previously (Toffoli et al., 2006). The GR was defined as the ratio of the SN38G AUC over the SN38 AUC. The BI was defined as the product of the irinotecan AUC and the ratio of the SN38 AUC over the SN38G AUC.

### Statistical Analysis

**FIGURE 1:**
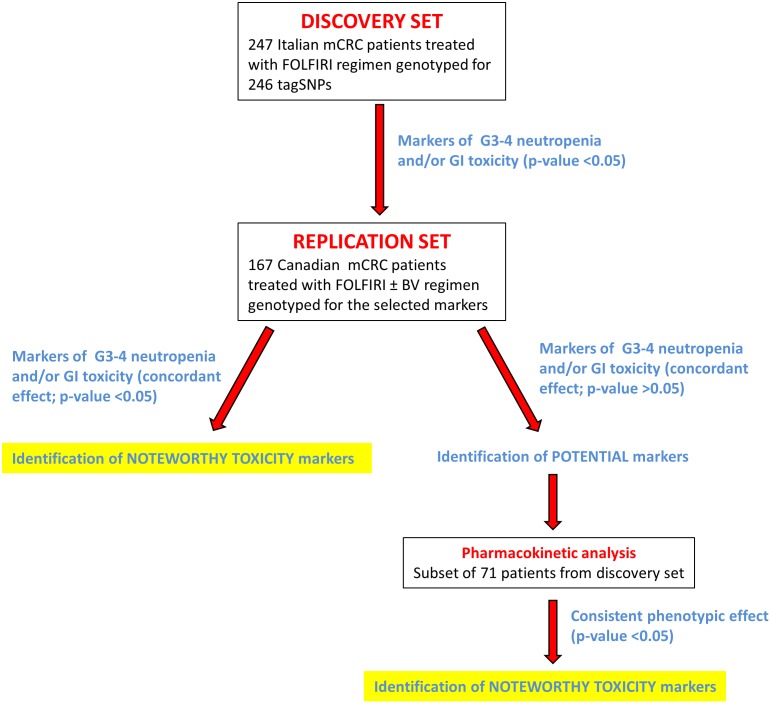
Flow chart summarizing the study design. More details are provided in the text. Abbreviations: BV, bevacizumab; G3–G4, grade 3–4; GI, gastrointestinal; mCRC, metastatic colorectal cancer; tagSNP, tagging polymorphism.

The overall study design is depicted in **Figure 1**. The analysis was carried out in three steps. The first step consisted of the selection of potential markers of severe toxicity (*p* < 0.05) in the discovery cohort. In the second step, these selected polymorphisms were independently tested in an independent cohort in order to find concordant associations (*p* < 0.05). Markers with a non-significant (*p* > 0.05) concordant effect (same genetic model, same effect) in both cohorts were still considered for possible association with irinotecan pharmacokinetic parameters, in a subgroup of patients from the discovery cohort.

Associations between genotype markers and outcome measures of FOLFIRI toxicity were assessed by multivariate logistic regression. Dominant, recessive, and additive genetic models were considered for each polymorphism by combining heterozygous and homozygous genotypes; the best-fitting genetic model was selected according to the Wald chi-square test. ORs and the corresponding 95% CIs were calculated and adjusted for the patients’ clinical and demographic characteristics available for each dataset (gender, age, first tumor site, stage at diagnosis, radical surgery, and adjuvant chemotherapy for the discovery cohort; gender, age, and co-treatment for the replication cohort). In all cases, significance was claimed for *p* < 0.05 (two-sided). To assess the effect of the multiple testing in the discovery cohort, a *q*-value [False discovery rate (FDR)-adjusted *p*-value] was evaluated (Benjamini and Hochberg, 1995). The distribution of pharmacokinetic parameters for irinotecan, SN-38, SN-38G, the BI, and GR according to genotype were compared by the Kruskal–Wallis test. All analyses were carried out in Stata 12.1.

## Results

### Genotyping

Genotyping was successful in 224/240 assays. BeadXpress technology failed at the analysis of 16 markers, which were successfully tested using the TaqMan method, as well as an additional six polymorphisms not included in the BeadXpress panel. The average genotype call rate was 0.99 (range: 0.90–1.00). Three of 250 samples were excluded from the study because they did not reach the fixed call rate threshold of 90%, probably due to low DNA quality; thus, genotype data were available for 247 patients that constituted the discovery set. Replicated samples included in the analyses presented an average concordance rate of 100%. Three randomly selected polymorphisms were used for analytical validation of the genotyping data using direct Sanger sequencing. Fifty samples were sequenced for *PXR* rs3732359, 49 for *RXRA* rs10881582, and 89 for *VDR* rs4237855. All of these markers had a concordance rate of 100%.

Analysis of the markers to be tested in the replication set, using the Sequenom platform was designed successfully for all candidate polymorphisms except rs880663, rs3093662, rs2744537, rs1800629, rs1800630, and rs2069840. All 167 samples constituting the replication set were positively genotyped; the average genotype call rate was 0.98 (range: 0.94–1.00).

### Patient Characteristics

The main demographic and clinical characteristics of the two study populations (discovery and replication cohorts) are reported in **Table 1**. All 247 genotyped patients were included in the cumulative toxicity evaluation (discovery cohort). The details of the toxicity assessment were previously reported (Toffoli et al., 2006). Severe neutropenia (grade 3–4) was present in 35 cases (14.2%) and corresponded to the most frequent severe toxicity. Diarrhea, nausea, and/or vomiting of high grade (grade 3–4) occurred in 26 cases (10.5%) and represented the predominant non-hematological toxicities. Among the 167 patients in the replication group, severe neutropenia was reported in 28 (16.8%), whereas grade 3–4 GI side effects were reported in 24 (14.4%).

**Table 1:** Demographic and clinical characteristics of the patients included in the study (discovery and replication cohort).

	Total (*n* = 414)	Italian cohort^∗^ (*n* = 247) *Discovery set*	Canadian cohort (*n* = 167) *Replication set*
	
Characteristic	*N* (%)	*N* (%)	*N* (%)
**Gender**			
Male	270 (65.2)	160 (64.8)	110 (65.87)
Female	144 (34.8)	87 (35.2)	57 (34.13)
**Age (years)**			
Median	62.9	63.5	62
**Primary tumor site**			
Colon	298 (72.0)	176 (71.3)	122 (73.1)
*Right*		*78 (44.3)*	*n.a*
*Left*		*98 (55.7)*	*n.a*
Rectum	113 (27.3)	71 (28.7)	42 (25.1)
Unknown	3 (0.7)	–	3 (1.8)
**Regimen**			
FOLFIRI	414 (100.0)	247 (100)	167 (100)
Co-treatment			
*Bevacizumab*		0	69 (41.3)
*Other drug*		0	6 (3.6)
**Toxicity**			
Neutropenia (grade 3–4)	63 (15.2)	35 (14.2)	28 (16.8)
Gastrointestinal (grade 3–4)	50 (12.1)	26 (10.5)	24 (14.4)


*^∗^A complete demographic and clinical description of Italian cohort was previously reported by Toffoli et al. (2006). The italic terms indicate a sub-category of the main characteristics. Abbreviations: n.a., not available.*

### Markers of Neutropenia

The association of each polymorphism with the occurrence of grade 3–4 neutropenia was tested by logistic regression analysis and the results summarized in **Table 2**. In the discovery cohort, 18 genetic variations in genes encoding four NRs (HNF4α, PXR, PPARs, and VDR), one transcription factor (NF-κB), and one cytokine (TNF) emerged as significant (*p* < 0.05) predictors of severe neutropenia over the entire course of chemotherapy. The genotype distribution based on neutropenia grade is reported in Supplementary Table S2; the MAFs of these polymorphisms were checked and found to be in line with the data reported for the Caucasian population^6^. Of the 18 markers identified, 10 were associated with an increased risk of developing grade 3–4 neutropenia, with ORs ranging from 1.68 to 16.08, and the remaining eight were indicated to have a protective effect against the development of toxicity, with ORs ranging from 0.16 to 0.60. Among the 18 markers associated with neutropenia at *p* < 0.05 in the discovery cohort, the *q*-value ranged from 0.181 to 0.505.

**Table 2:** Odds ratios (ORs) and 95% confidence interval (CI) for grade 3–4 vs. grade 0–2 cumulative neutropenia in the discovery (*n* = 247 mCRC patients) and replication (*n* = 167 mCRC patients) cohorts according to gene polymorphisms (SNPs).

Discovery set						Replication set		
	
Genes	SNP	Base Change	Model	OR (95% CI)^a^	*p*-value	Model	OR (95% CI)^b^	*p*-value
*HNF4A*	rs2425637	G > T	Additive	1.88 (1.05–3.38)	0.035	Dominant	1.51 (0.53–4.27)	0.442
*HNF4A*	rs3212183	T > C	Recessive	2.50 (1.11–5.56)	0.026	Additive	1.20 (0.65–2.21)	0.558
*HNF4A*	rs3212197	C > T	Additive	2.29 (1.07–4.91)	0.033	Dominant	0.47 (0.09–2.35)	0.357
***HNF4A***	**rs6093976**	**C > T**	**Dominant**	**0.16 (0.05–0.56)**	**0.004**	**Dominant**	**0.67 (0.27–1.64)**	**0.379**
*HNF4A*	rs6093978	C > T	Dominant	0.41 (0.18–0.93)	0.033	Recessive	1.83 (0.59–5.68)	0.292
*HNF4A*	rs6130615	C > T	Recessive	16.08 (2.89–89.62)	0.002	Additive	0.70 (0.27–1.79)	0.457
***HNF4A***	**rs745975**	**G > A**	**Dominant**	**0.33 (0.13–0.85)**	**0.021**	**Dominant**	**0.68 (0.29–1.61)**	**0.380**
*HNF4A*	rs2425640	G > A	Additive	0.60 (0.36–0.98)	0.042	Recessive	3.35 (0.82–13.66)	0.092
*NR1I2*	rs16830505	A > G	Dominant	2.33 (1.01–5.34)	0.046	Additive	1.87 (0.83–4.23)	0.132
*NR1I2*	rs7643645	A > G	Dominant	0.28 (0.13–0.61)	0.001	Recessive	3.01 (1.01–9.01)	0.049
*PPARD*	rs2076169	T > C	Recessive	8.99 (1.31–61.85)	0.026	Dominant	0.18 (0.02–1.42)	0.103
*PPARG*	rs2972164	T > C	Dominant	0.36 (0.16–0.82)	0.015	Dominant	1.34 (0.49–3.69)	0.565
*PPARG*	rs880663	T > C	Additive	0.36 (0.16–0.82)	0.015	–	–	–
*NFKB1*	rs230539	A > G	Additive	1.68 (1.02–2.78)	0.043	Recessive	0.42 (0.09–1.87)	0.254
*TNF*	rs3093662	A > G	Dominant	2.56 (1.10–5.96)	0.029	–	–	–
*VDR*	rs11168287	A > G	Dominant	3.12 (1.02–9.56)	0.046	Recessive	1.98 (0.73–5.43)	0.182
*VDR*	rs11574026	C > T	Dominant	2.36 (1.06–5.23)	0.035	Recessive	1.55 (0.63–3.77)	0.339
***VDR***	**rs12717991**	**G > A**	**Dominant**	**0.36 (0.16–0.82)**	**0.015**	**Dominant**	**0.84 (0.35–2.00)**	**0.695**


*Only the associations with p-value* < *0.05 are reported in the discovery set; markers with the same predictive effect and genetic model in both cohort are evidenced in bold. ^*a*^Estimated from unconditional logistic regression, adjusted for gender, age, cancer site, stage at diagnosis, radical surgery, and adjuvant chemotherapy. ^*b*^Estimated from unconditional logistic regression, adjusted for gender, age, co-treatment. Abbreviations: mCRC, metastatic colorectal cancer*.

None of these associations was significant in the replication set (*p* > 0.05).

### Markers of GI Toxicity

The association of each polymorphism with the occurrence of grade 3–4 GI toxicity was tested by logistic regression analysis and the data reported in **Table 3**. Twenty-two polymorphic variants in genes encoding five NRs (HNF4α, CAR, PPARs, RXRs, and VDR), one transcription factor (STAT-3), and three cytokines (TNF, IFN-γ, and IL-6) were significantly (*p* < 0.05) associated with the risk of developing severe GI toxicity during the entire course of chemotherapy. The genotype distribution by GI toxicity grade is reported in Supplementary Table S3; the MAFs of these polymorphisms were in line with the data reported for the Caucasian population (see foot note text 6). Of the 22 markers identified, 14 were associated with an increased chance of having grade 3–4 GI toxicity, with ORs ranging from 1.72 to 20.74, and the remaining eight correlated with an inferior risk of severe GI toxicity, with ORs ranging from 0.12 to 0.51. Among the 22 markers associated with GI toxicity at *p* < 0.05 in the discovery cohort, the *q*-value ranged from 0.244 to 0.521.

**Table 3:** Odds ratios (ORs) and 95% confidence interval (CI) for grade 3–4 vs. grade 0–2 cumulative gastrointestinal toxicity in the discovery (*n* = 247 mCRC patients) and replication (*n* = 167 mCRC patients) cohort according to gene polymorphisms (SNPs).

Discovery set						Replication set		
	
Genes	SNP	Base Change	Model	OR (95% CI)^a^	*p*-value	Model	OR (95% CI)^b^	*p*-value
***HNF4A***	**rs1800961**	**C > T**	**Dominant**	**11.51 (2.02–65.61)**	**0.006**	**Dominant**	**3.27 (0.57–18.69)**	**0.182**
***HNF4A***	**rs2071197**	**G > A**	**Dominant**	**4.42 (1.75–11.17)**	**0.002**	**Dominant**	**1.43 (0.45–4.58)**	**0.546**
***HNF4A***	**rs6031587**	**C > T**	**Additive**	**3.24 (1.40–7.51)**	**0.006**	**Additive**	**2.21 (0.86–5.70)**	**0.100**
*HNF4A*	rs6093976	C > T	Additive	0.36 (0.13–0.98)	0.046	Recessive	1.92 (0.35–10.65)	0.457
*NR1I3*	rs2307424	C > T	Dominant	3.38 (1.23–9.27)	0.018	Additive	0.66 (0.35–1.24)	0.196
*NR1I3*	rs4073054	T > G	Recessive	0.14 (0.02–0.92)	0.041	Dominant	0.63 (0.26–1.50)	0.293
*PPARA*	rs9626736	A > G	Additive	2.14 (1.14–4.00)	0.018	Dominant	1.90 (0.71–5.08)	0.203
***PPARD***	**rs2076169**	**T > C**	**Recessive**	**20.74 (2.88–149.18)**	**0.003**	**Recessive**	**6.26 (0.36–109.01)**	**0.208**
***RXRG***	**rs380518**	**T > C**	**Dominant**	**4.69 (1.73–12.70)**	**0.002**	**Dominant**	**2.15 (0.82–5.66)**	**0.120**
*RXRG*	rs4657437	C > A	Additive	0.48 (0.24–0.99)	0.047	Dominant	2.54 (0.90–7.22)	0.080
*RXRG*	rs283695	G > A	Additive	1.80 (1.04–3.12)	0.036	Recessive	0.43 (0.10–1.98)	0.282
***RXRG***	**rs3767344**	**G > C**	**Dominant**	**0.35 (0.13–0.93)**	**0.034**	**Dominant**	**0.75 (0.28–2.03)**	**0.574**
*RXRG*	rs157880	C > T	Recessive	11.70 (20.03–67.58)	0.006	Dominant	1.44 (0.58–3.59)	0.435
*RXRB*	rs2744537	G > T	Recessive	5.34 (1.42–20.07)	0.013	–	–	–
***VDR***	**rs11574077**	**A > G**	**Additive**	**4.46 (1.43–13.96)**	**0.010**	**Additive**	**1.44 (0.37–5.63)**	**0.601**
*VDR*	rs4760648	C > T	Additive	2.09 (1.13–3.84)	0.018	Additive	0.71 (0.36–1.42)	0.338
*VDR*	rs2853564	T > C	Additive	0.38 (0.18–0.78)	0.008	Additive	1.42 (0.72–2.78)	0.310
*TNF*	rs1800629	A > G	Dominant	4.07 (1.13–14.72)	0.032	–	–	–
*TNF*	rs1800630	C > A	Additive	1.72 (1.01–2.94)	0.047	–	–	–
***STAT3***	**rs1053004**	**T > C**	**Additive**	**0.51 (0.27–0.99)**	**0.045**	**Dominant**	**0.39 (0.15–0.97)**	**0.043**
***INFG***	**rs2069716**	**A > G**	**Dominant**	**0.12 (0.01–0.97)**	**0.047**	**Dominant**	**0.88 (0.20–3.88)**	**0.862**
*IL6*	rs2069840	C > G	Dominant	0.40 (0.17–0.95)	0.039	–	–	–


*Only the associations with p-value* < *0.05 are reported in the discovery set; markers with the same predictive effect and genetic model in both cohort are evidenced in bold. Replicated markers are underlined. ^*a*^Estimated from unconditional logistic regression, adjusted for gender, age, cancer site, stage at diagnosis, radical surgery, and adjuvant chemotherapy. ^*b*^Estimated from unconditional logistic regression, adjusted for gender, age, co-treatment. Abbreviations: mCRC, metastatic colorectal cancer*.

One *STAT-3* marker (rs1053004) presented a concordant significant effect in the replication cohort (*p* < 0.05) as a protective factor against the development of grade 3–4 GI toxicity. The C allele at *STAT-3* rs1053004 was significantly associated with a decreased risk of toxicity in the discovery cohort (OR = 0.51, *p* = 0.045, *q* = 0.521) according to an additive model. Similarly, *STAT-3* rs1053004 C allele exerted a protective effect against the development of toxicity in the replication cohort (OR = 0.39, *p* = 0.043, *q* = 0.305) in a dominant model. None of the other associations between genetic markers and the risk of severe GI toxicity found in the discovery cohort were significant in the replication cohort (*p* > 0.05).

### Pharmacokinetics

**FIGURE 2:**
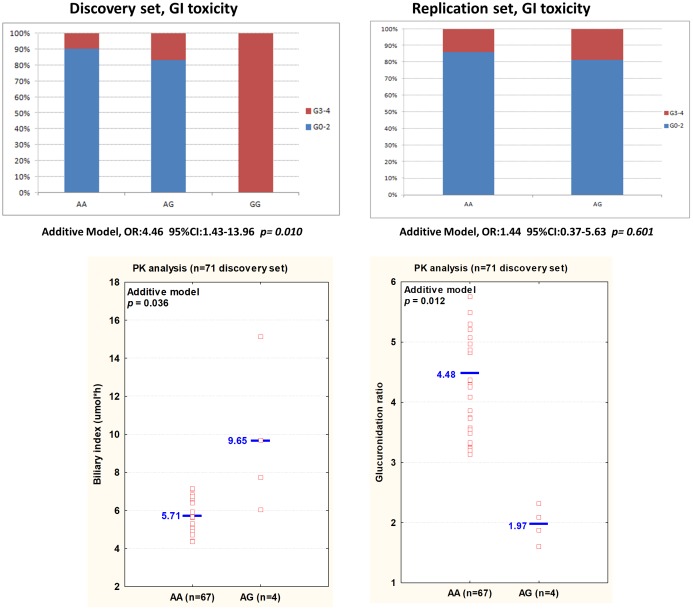
**(Top)** An histogram plot displays the percentage of patients experiencing mild (grade 0–2) or severe (grade 3–4) GI toxicity, according to *VDR* rs11574077 genotype in the discovery and replication sets. **(Bottom)** The biliary index and glucuronidation ratio values for each patient, according to *VDR* rs11574077 genotype, are shown. The analysis has been performed on 71 patients from the discovery set. Each dot represents a patient. Abbreviations: 95%CI, 95% confidence intervals; AUC, area under the curve; BI, biliary index [CPT-11 AUC X (SN38 AUC/SN38G AUC)]; CPT-11, irinotecan; GI, gastrointestinal toxicity; GR, glucuronidation ratio (SN-38G AUC/SN-38 AUC); OR, Odds ratios; SN-38, active form of irinotecan; PK, pharmacokinetic; SN-38G, inactive glucuronidation form of irinotecan.

The pharmacokinetic analysis focused on markers that, even if not presenting a significant effect in the replication cohort (*p* > 0.05), presented a concordant effect on the toxicity risk in both cohorts (same size effect according to the same genetic model, **Tables 2**, **3**). The association of these polymorphisms with the pharmacokinetic parameters was investigated in a subset of 71 patients from the discovery cohort, and the most relevant results (*p* < 0.1; concordant genetic model) summarized in Supplementary Table S4. *VDR* rs11574077 was associated to an inferior GR (*p* = 0.012) and an increased BI (*p* = 0.036) according to an additive model (**Figure 2**). None of the four patients harboring the rs11574077-G variant allele were homozygous for *UGT1A^∗^*28 polymorphism, excluding a potential confounding effect. The pharmacokinetic correlations with the other polymorphisms analyzed were not significant but still presented an effect consistent with that reported on the toxicity risk. *HNF4A* rs6031587 correlated with an increased AUC for irinotecan (*p* = 0.091), whereas *VDR* rs12717991 (*p* = 0.073) and *RXRG* rs3767344 (*p* = 0.078) were associated with an increased GR consistent with the hypothesized protective effect against the development of toxicity.

## Discussion

Despite the introduction of targeted agents in the pharmacological treatment of mCRC, combinatorial chemotherapy regimens are still the backbone of each therapeutic scheme, burdening the patients with unpredictable adverse events. Neutropenia and GI toxicity are typically associated with irinotecan-containing regimens, such as FOLFIRI (Gupta et al., 1994, 1997). In the last few years, several pharmacogenetic studies have sought to identify predictive genetic markers impacting irinotecan ADME genes and, consequently, its pharmacokinetics and pharmacodynamics in order to help clinicians personalize irinotecan-based therapy. Despite these efforts, the only validated predictor of severe toxicity is currently *UGT1A1*^∗^28 polymorphism, adopted by international guidelines for adjusting the irinotecan dose (Swen et al., 2011; Caudle et al., 2013), which does not completely address the issue. Additional work is required to define further molecular markers and better identify patients at risk for severe complications. A more recent field of investigation is the contribution of genetic variability in specific transcriptional regulators encoding genes to the impact on the control of ADME gene expression (Ho and Piquette-Miller, 2006; Reuter et al., 2010; De Mattia et al., 2013b, 2016; Cecchin et al., 2016). The main findings of the present study were the identification of *STAT-3* rs1053004 polymorphism as a promising marker of grade 3–4 GI toxicity, and of *VDR* rs11574077 as a marker of irinotecan pharmacokinetics with a consistent effect on grade 3–4 GI toxicity risk. An independent cohort of FOLFIRI-treated mCRC patients was adopted to independently replicate these results.

The IL-6/STAT-3 cascade has been indirectly implicated in the regulation of key irinotecan pathway-related proteins, such as cytochromes (i.e., CYP3A4) (Jover et al., 2002) and ABC/SLC transporters (i.e., MDR-1) (Duan et al., 2006; Ho and Piquette-Miller, 2006; Andrejko et al., 2008; Zhang et al., 2011; Zhu et al., 2012), which play a crucial role in drug disposition. In this respect, STAT-3 could mediate the impact of CRC-associated inflammation (Markman and Shiao, 2015) on irinotecan-related ADME gene expression, affecting the toxicity profile (Chen et al., 2012; Cressman et al., 2012; Harvey and Morgan, 2014). STAT-3 is triggered in response to the binding of numerous pro-inflammatory cytokines and represents the critical factor in IL-6-induced gene control (Aggarwal et al., 2009; Qi and Yang, 2014; Yu et al., 2014). Once activated, STAT-3 transduces the signal across the cytoplasm and into the nucleus, where it mediates gene transcription, often in cooperation with NF-κB (Aggarwal et al., 2009; Qi and Yang, 2014; Yu et al., 2014). The *STAT-3* rs1053004 polymorphism located in the 3′ untranslated region of the gene in a putative binding site for miR-423-5p has been reported to regulate the expression of STAT-3 protein, probably by altering mRNA degradation, and to significantly affect its transcriptional activity (Permuth-Wey et al., 2016). The altered STAT-3 activity associated with this polymorphic variant could potentially contribute to altering the bioavailability of irinotecan and, consequently, the individual predisposition to experience severe GI side effects. As a mediator of cytokine signaling, STAT-3 could also be directly involved in the complex mechanism underlying the toxic damage to the GI epithelium (i.e., mucositis) induced by some chemotherapeutics, including irinotecan. The pathobiology of drug-related mucositis involves the mucosal immune system, with pro-inflammatory cytokine release playing an important role (Logan et al., 2008; Lee et al., 2014). In particular, this mechanism has been implicated in the onset of intestinal injury subsequent to the administration of irinotecan (Logan et al., 2008; Melo et al., 2008), as well 5-fluorouracil, the other drug included in the FOLFIRI regimen (Lee et al., 2014). Thus, IL-6/STAT-3 signaling could contribute to FOLFIRI-related mucosal damage by controlling the proliferation and survival of intestinal epithelial cells as observed in colitis (Moriasi et al., 2012; Nguyen et al., 2015). Furthermore, an interaction between the IL-6/STAT-3 cascade and the gut microbioma, which contributes to the local accumulation of the cytotoxic active metabolite SN-38 (Mathijssen et al., 2001), has been described (Fichtner-Feigl et al., 2015; Jurjus et al., 2016). Therefore, a change in the STAT-3 activity could significantly alter the mediation of pro-inflammatory cytokines, such as IL-6, IL-1β, and TNF-α, modifying the susceptibility of the GI mucosa to the toxic effect of irinotecan-based treatment as shown in the present study. A significant effect of some germ line genetic variations in these cytokines (i.e., IL-6, and TNFα) was demonstrated in the discovery cohort (**Table 3**). Unfortunately, the missing genotype data for the replication cohort did not permit to independently replicate their effect.

Concerning *VDR* rs11574077 polymorphism, it was found to significantly correlate with an increased BI and inferior GR, parameters indicating lower efficacy of the glucuronidation and detoxification pathways. This functional effect of the polymorphism on the pharmacokinetics of irinotecan is consistent with its detrimental impact on the occurrence of grade 3–4 GI toxicity, though it was not significantly replicated in the Canadian cohort, possibly due to the lower number of patients in this group and the low MAF of the polymorphism. Beyond its physiological role in calcium and phosphate homeostasis, VDR has been demonstrated to cooperate in the transcriptional regulation of ADME genes (i.e., *CYPs, UGT1As, ABC/SLC* transporters) (Prakash et al., 2015), possibly affecting the irinotecan disposition profile. Thus, *VDR* rs11574077 represents an intronic variant of unknown functional significance, although it was reported to impact the risk of developing some tumors and cardiovascular disease (Verschuren et al., 2012; Muindi et al., 2013). Though the exact molecular mechanism of *VDR* expression/activity regulation by rs1799794 variant is currently unclear, and the hypothesis that its effect could be due to linkage disequilibrium with another functional marker cannot be excluded. However, the potentially altered VDR activity due to the polymorphism could influence drug bioavailability with significant consequences on the irinotecan-based modulation of GI toxicity.

Some of the genetic markers selected in the first step of the statistical analysis in the discovery cohort were not confirmed in the replication cohort. Other markers presented a concordant, but not significant, effect in the replication cohort (**Tables 2**, **3**). It must be kept in mind that some inhomogeneity existed between the two cohorts and we cannot exclude an ethnicity-specific effect of some of the analyzed genetic variants. For these markers, additional replication studies will be required to elucidate their possible role in determining the irinotecan toxicity risk.

Some limitations of the present study need to be considered. Firstly, the FOLFIRI regimen is no longer the standard therapy for mCRC patients, but it is still given in combination with monoclonal antibodies. A subset of the patients in the replication cohort was treated with a combination of FOLFIRI and bevacizumab, supporting a predictive effect of the markers in such poly-chemotherapy-treated patients. Secondly, the functional meanings of the markers highlighted in the present study are unknown. Formal functional analyses should be performed in order to better understand the molecular mechanism underlying the observed associations, but the concordant pharmacokinetic effect found for some of the highlighted markers supports a functional effect of the variant on the efficiency of the enzymatic activity of the encoded protein. Thirdly, the present study focused only on common genetic variants with a MAF ≥ 0.05. As recently pointed out, rarer genetic variants could account for a high percentage of inter-individual variability in drug metabolism, including NR genes (Lauschke and Ingelman-Sundberg, 2016; Kozyra et al., 2017), and for the observed inter-individual heterogeneity in drug toxicity and pharmacokinetics. Therefore, future pharmacogenetic approaches should include these emerging markers in order to better describe patient phenotypes regarding the response to pharmacological treatment. It must be noticed that, when controlling the analysis for multiple testing, all the associations were above 18.1% of FDR, pointing out that the study results must be considered only as hypothesis-generating. It is nonetheless acknowledged that replicating a significant association in an independent set of patients, as in the present study, strengthens the reliability of the data and the interest to further clarify their potential clinical implication.

## Conclusion

The present study suggested for the first time that the *STAT-3* rs1053004 polymorphism could have an effect on the risk of severe GI toxicity after irinotecan therapy (FOLFIRI regimen) in mCRC patients. This finding further highlights the importance of inflammatory response mediators in the pathobiology of drug-induced mucosal injury, a topic that requires more attention in pharmacogenetic investigations. *VDR* rs11574077 also emerged as a promising novel determinant of irinotecan pharmacokinetic parameters and possibly of FOLFIRI-related GI toxicity risk, soliciting future research efforts in this direction. The discovery of novel factors contributing to the individual predisposition to the development of severe GI toxicity after chemotherapy is of great interest considering the increased recognition of the clinical and economic implications of the mucosal damage induced by treatment (Gibson et al., 2015). These findings could help elucidate the molecular mechanism underlying irinotecan-induced epithelial injury and, if validated, could be considered as additional criteria to improve the clinical management of FOLFIRI-related GI toxicity.

## Author Contributions

EDM and EC contributed equally to designing the study, writing the main manuscript, and elaborating the tables and figures. MM was involved in the statistical analysis and interpretation of data. AL participated in the molecular analysis for replication cohort. CG participated in the enrollment of the replication patient group and the collection of clinical and genotyping data on this set. ED, RR, and AlB participated in the molecular analysis for the discovery cohort. AnB, MD’A, LC, and SL participated in the enrollment of the discovery patient group and the collection of clinical data. EL, DJ, and FC participated in the design, enrolment of the replication cohort, and the collection of clinical data. GT was the guarantor. All authors reviewed the manuscript.

## Conflict of Interest Statement

The authors declare that the research was conducted in the absence of any commercial or financial relationships that could be construed as a potential conflict of interest.

## Footnotes

1 http://hapmap.ncbi.nlm.nih.gov/

2 http://www.broad.mit.edu/mpg/haploview

3 http://www.ncbi.nlm.nih.gov/

4 https://www.illumina.com/

5 www.appliedbiosystems.com

6 http://www.ncbi.nlm.nih.gov/snp

## Abbreviations

ABC: ATP-binding cassette
ADME: adsorption, distribution, metabolism and excretion
AUC: area under the curve
BI: biliary index
CIs: corresponding 95% confidence intervals
CPT-11: irinotecan
FOLFIRI: irinotecan, 5-fluorouracil and leucovorin
GI: gastrointestinal
GR: glucuronidation ratio
MAFs: minor allele frequencies
mCRC: metastatic colorectal cancer
ORs: odds ratios
SLC: solute carrier
TagSNP: tagging polymorphism
UGT1A: UDP-glucuronosyltransferase 1A
