# Supplementary material for: Association of STAT-3 rs1053004 and VDR rs11574077 With FOLFIRI-Related Gastrointestinal Toxicity in Metastatic Colorectal Cancer Patients
Source: Front Pharmacol. 2018 Apr 13;9:367. doi: 10.3389/fphar.2018.00367 (PMC5908896; doi:10.3389/fphar.2018.00367)
Supplement: Supplementary file 1 [file Table_1.doc]

Supplementary Material

Title: *STAT-3* rs1053004 and *VDR* rs11574077 as predictors of gastrointestinal toxicity in metastatic colorectal cancer patients receiving first-line FOLFIRI treatment.

**Authors:** Elena De Mattia^1§^, Erika Cecchin^1§^, Marcella Montico^1^, Adrien Labriet^2^, Chantal Guillemette^2.3^, Eva Dreussi^1^, Rossana Roncato^1^, Alessia Bignucolo^1^, Angela Buonadonna^4^, Mario D’Andrea^5^, Luigi Coppola^6^, Sara  Lonardi^7^, Eric Lévesque^8^, Derek Jonker^9^, Félix Couture^8^, Giuseppe Toffoli^1*^

**Correspondence to:**

***Dr. Giuseppe Toffoli MD, Director,** Clinical and Experimental Pharmacology, CRO- National Cancer Institute, Via Franco Gallini n. 2, 33081 Aviano (PN) –Italy. [gtoffoli@cro.it](mailto:gtoffoli@cro.it)

Telephone +39-0434-659612 and Fax +39-0434-659799

**Supplementary Table S1:** Candidate genes and related TagSNPs/polymorphisms selected for pharmacogenetic analysis.

| GENE | TagSNPs (exonic/intronic region) | SNPs (5’/3’ UTR) |
| --- | --- | --- |
| *PXR; NR1I2* | rs1403527 |  |
|  | rs13059232 |  |
|  | rs3814055 |  |
|  | rs1403526 |  |
|  | rs3732357 |  |
|  | rs11929668 |  |
|  | rs16830505 |  |
|  | rs3814057 |  |
|  | rs6784598 |  |
|  | rs3732359 |  |
|  | rs3732360 |  |
|  | rs1054190 |  |
|  | rs7643645 |  |
| *FXR; NR1H4* | rs35724 |  |
|  | rs1030454 |  |
|  | rs11110415 |  |
|  | rs11110390 |  |
|  | rs11610264 |  |
|  | rs17030285 |  |
|  | rs4764980 |  |
| *RXR-A; NR2B1* | rs4917353 |  |
|  | rs3118536 |  |
|  | rs7864987 |  |
|  | rs10881582 |  |
|  | rs1805352 |  |
|  | rs877954 |  |
|  | rs11103482 |  |
|  | rs3132294 |  |
|  | rs11185659 |  |
|  | rs7038018 |  |
|  | rs4240705 |  |
|  | rs1045570 |  |
|  | rs11103473 |  |
|  | rs6537944 |  |
|  | rs10776909 |  |
|  | rs7039190 |  |
| *RXR-B; NR2B2* | rs2744537 |  |
|  | rs2072915 |  |
|  | rs2076310 |  |
| *RXR-G; NR2B3* | rs157864 |  |
|  | rs157869 |  |
|  | rs3767344 |  |
|  | rs3767333 |  |
|  | rs157880 |  |
|  | rs10800098 |  |
|  | rs3767339 |  |
|  | rs4657437 |  |
|  | rs10489747 |  |
|  | rs285480 |  |
|  | rs285481 |  |
|  | rs1123944 |  |
|  | rs380518 |  |
|  | rs746332 |  |
|  | rs2651860 |  |
|  | rs10489745 |  |
|  | rs100537 |  |
|  | rs283690 |  |
|  | rs752739 |  |
|  | rs283695 |  |
|  | rs157862 |  |
|  | rs285482 |  |
|  | rs283694 |  |
| *LXR-A;* [*NR1H3*](http://www.ncbi.nlm.nih.gov/gene/10062) | rs7120118 | rs11039149 |
|  |  | rs10838681 |
| *LXR-B; NR1H2* | rs1405655 | rs4802703 |
| *CAR;* [*NR1I3*](http://www.ncbi.nlm.nih.gov/gene/9970) | rs2501873 |  |
|  | rs4073054 |  |
|  | rs2307424 |  |
|  | rs2307418 |  |
|  | rs6686001 |  |
| *VDR; NR1I1* | rs2248098 | rs11574143 |
|  | rs11168292 | rs7139166 |
|  | rs10875695 | rs4516035 |
|  | rs757343 | rs11568820 |
|  | rs2239180 |  |
|  | rs11574012 |  |
|  | rs1544410 |  |
|  | rs10783219 |  |
|  | rs886441 |  |
|  | rs12717991 |  |
|  | rs3782905 |  |
|  | rs2853564 |  |
|  | rs2239182 |  |
|  | rs11574046 |  |
|  | rs2107301 |  |
|  | rs2238136 |  |
|  | rs3819545 |  |
|  | rs2239186 |  |
|  | rs4328262 |  |
|  | rs11574026 |  |
|  | rs7299460 |  |
|  | rs11168287 |  |
|  | rs11168275 |  |
|  | rs4760648 |  |
|  | rs2189480 |  |
|  | rs2239179 |  |
|  | rs2254210 |  |
|  | rs11574077 |  |
| *PPAR-A; NR1C1* | rs14842 |  |
|  | rs5766743 |  |
|  | rs6008259 |  |
|  | rs135551 |  |
|  | rs4253711 |  |
|  | rs135547 |  |
|  | rs135538 |  |
|  | rs11703495 |  |
|  | rs9627046 |  |
|  | rs4253701 |  |
|  | rs4253623 |  |
|  | rs9615264 |  |
|  | rs12330015 |  |
|  | rs6007662 |  |
|  | rs9626736 |  |
|  | rs1555208 |  |
|  | rs6008197 |  |
|  | rs4253662 |  |
|  | rs11090819 |  |
|  | rs4253655 |  |
|  | rs4253755 |  |
| *PPAR-D; NR1C2* | rs9658100 |  |
|  | rs2076167 |  |
|  | rs2016520 |  |
|  | rs7744392 |  |
|  | rs9470001 |  |
|  | rs9658119 |  |
|  | rs4713854 |  |
|  | rs1053046 |  |
|  | rs2076169 |  |
| *PPAR-G; NR1C3* | rs880663 |  |
|  | rs1175540 |  |
|  | rs1801282 |  |
|  | rs2938392 |  |
|  | rs4135284 |  |
|  | rs17793951 |  |
|  | rs17036281 |  |
|  | rs13099828 |  |
|  | rs7626560 |  |
|  | rs4135275 |  |
|  | rs2120825 |  |
|  | rs2972164 |  |
|  | rs3856806 |  |
|  | rs4135268 |  |
|  | rs4135247 |  |
|  | rs1797912 |  |
| *HNF4A; NR2A1* | rs3212198 | rs6130615 |
|  | rs6031551 |  |
|  | rs11574738 |  |
|  | rs8116574 |  |
|  | rs2425637 |  |
|  | rs6065725 |  |
|  | rs4812829 |  |
|  | rs2868094 |  |
|  | rs3212208 |  |
|  | rs6093978 |  |
|  | rs3212183 |  |
|  | rs745975 |  |
|  | rs717248 |  |
|  | rs6031595 |  |
|  | rs3092370 |  |
|  | rs6031587 |  |
|  | rs2425640 |  |
|  | rs6093976 |  |
|  | rs4364072 |  |
|  | rs11574736 |  |
|  | rs6103716 |  |
|  | rs8114057 |  |
|  | rs2071200 |  |
|  | rs6031580 |  |
|  | rs11574730 |  |
|  | rs4812831 |  |
|  | rs3818247 |  |
|  | rs3212197 |  |
|  | rs1800961 |  |
|  | rs6017335 |  |
|  | rs2071197 |  |
|  | rs6073418 |  |
|  | rs11574733 |  |
| *HNF1A* | rs2244608 |  |
|  | rs2393791 |  |
|  | rs1169293 |  |
|  | rs1169300 |  |
|  | rs1169302 |  |
|  | rs1169286 |  |
|  | rs1169307 |  |
|  | rs1882149 |  |
|  | rs3999413 |  |
|  | rs1169303 |  |
|  | rs12427353 |  |
|  | rs735396 |  |
|  | rs2071190 |  |
| *STAT-3* | rs1026916 |  |
|  | rs1053005 |  |
|  | rs12949918 |  |
|  | rs17593222 |  |
|  | rs6503695 |  |
|  | rs1053004 |  |
|  | rs8069645 |  |
| *NFκB1* | rs230539 |  |
|  | rs230496 |  |
|  | rs4647992 |  |
|  | rs4648110 |  |
|  | rs11722146 |  |
|  | rs4648135 |  |
|  | rs3774934 |  |
|  | rs4648022 |  |
|  | rs1598861 |  |
|  | rs4648127 |  |
|  | rs4648090 |  |
| [*IKBKB*](http://www.ncbi.nlm.nih.gov/gene/3551) | rs6474388 |  |
|  | rs2272733 |  |
|  | rs9694958 |  |
|  | rs17875671 |  |
|  | rs5029748 |  |
|  | rs3747811 |  |
|  | rs10958713 |  |
| *CHUK* | rs3818411 |  |
|  | rs11591741 |  |
|  | rs12570957 |  |
|  | rs11595324 |  |
|  | rs3818411 |  |
|  | rs11190430 |  |
| *IL-1B* | rs1143643 | rs1143627 |
|  | rs3136558 | rs1143623 |
|  | rs1143634 | rs16944 |
| *IL-6* | rs2069845 | rs2069861 |
|  | rs2069840 | rs2069827 |
|  |  | rs1800797 |
|  |  | rs1800795 |
| *INFG* | rs1861494 | rs2069727 |
|  | rs2069716 |  |

Abbrevations: 3/5’-UTR; 3’/5’ untranslated region defined according to Genomic Build: hg19/GRCh37 (Feb 2009); *CAR*, constitutive androstane receptors (NR1I3); [*CHUK*](http://www.ncbi.nlm.nih.gov/gene/1147), conserved helix-loop-helix ubiquitous kinase; *FXR*, farnesoid X receptor; *HNF1A*, HNF1 homeobox A; *HNF4A*; hepatocyte nuclear factors 4 (NR2A1); *IL-1B*, interleukin-1 beta; *IL-6*, interleukin-6; *INFG*, interferon gamma; [*IKBKB*](http://www.ncbi.nlm.nih.gov/gene/3551), inhibitor of kappa light polypeptide gene enhancer in B-cells, kinase beta, *LXR-A,B*, liver X receptors (NR1H3 and NR1H2); *NFκB1*, nuclear factor κ B; *PPAR-A,D,G*, peroxisome proliferator-activated receptors (NR1C1, NR1C2 and NR1C3), *PXR*, pregnane X receptor; *RXR-A,B,C*, retinoid X receptors (RXR; NR2B1, NR2B2 and NR2B3); *STAT-3*, signal transducers and activators of transcription; TagSNPs: tagging polymorphisms; *TNF*, tumor necrosis factor; *VDR*, vitamin D receptor (NR1I1).
