# Supplementary material for: Association of STAT-3 rs1053004 and VDR rs11574077 With FOLFIRI-Related Gastrointestinal Toxicity in Metastatic Colorectal Cancer Patients
Source: Front Pharmacol. 2018 Apr 13;9:367. doi: 10.3389/fphar.2018.00367 (PMC5908896; doi:10.3389/fphar.2018.00367)
Supplement: Supplementary file 2 [file Table_2.doc]

Supplementary Material

Title: *STAT-3* rs1053004 and *VDR* rs11574077 as predictors of gastrointestinal toxicity in metastatic colorectal cancer patients receiving first-line FOLFIRI treatment.

**Authors:** Elena De Mattia^1§^, Erika Cecchin^1§^, Marcella Montico^1^, Adrien Labriet^2^, Chantal Guillemette^2.3^, Eva Dreussi^1^, Rossana Roncato^1^, Alessia Bignucolo^1^, Angela Buonadonna^4^, Mario D’Andrea^5^, Luigi Coppola^6^, Sara  Lonardi^7^, Eric Lévesque^8^, Derek Jonker^9^, Félix Couture^8^, Giuseppe Toffoli^1*^

**Correspondence to:**

***Dr. Giuseppe Toffoli MD, Director,** Clinical and Experimental Pharmacology, CRO- National Cancer Institute, Via Franco Gallini n. 2, 33081 Aviano (PN) –Italy. [gtoffoli@cro.it](mailto:gtoffoli@cro.it)

Telephone +39-0434-659612 and Fax +39-0434-659799

**Supplementary Table S2**. **Genotype frequency distribution for grade 3-4 vs. grade 0-2 cumulative neutropenia in the discovery set (n=247 mCRC patients) according to gene polymorphisms.**

| **Genes** | **SNP** | **Base change** | **Genotype frequency** | | | | | | |
| --- | --- | --- | --- | --- | --- | --- | --- | --- | --- |
|  |  |  | **grade 0-2 (n=212)** | | |  | **grade 3-4 (n=35)** | | |
|  |  |  | **AA** | **Aa** | **aa** |  | **AA** | **Aa** | **aa** |
| *HNF4A* | rs2425637 | G>T | 0.269 | 0.509 | 0.222 |  | 0.171 | 0.429 | 0.400 |
| *HNF4A* | rs3212183 | T>C | 0.332 | 0.455 | 0.213 |  | 0.229 | 0.371 | 0.400 |
| *HNF4A* | rs3212197 | C>T | 0.852 | 0.143 | 0.005 |  | 0.771 | 0.143 | 0.086 |
| *HNF4A* | rs6093976 | C>T | 0.632 | 0.335 | 0.033 |  | 0.886 | 0.114 | 0.000 |
| *HNF4A* | rs6093978 | C>T | 0.445 | 0.403 | 0.152 |  | 0.657 | 0.257 | 0.086 |
| *HNF4A* | rs6130615 | C>T | 0.741 | 0.250 | 0.009 |  | 0.657 | 0.257 | 0.086 |
| *HNF4A* | rs745975 | G>A | 0.561 | 0.401 | 0.038 |  | 0.771 | 0.200 | 0.029 |
| *HNF4A* | rs2425640 | G>A | 0.434 | 0.415 | 0.151 |  | 0.543 | 0.429 | 0.028 |
| *NR1I2* | rs16830505 | A>G | 0.816 | 0.170 | 0.014 |  | 0.686 | 0.286 | 0.028 |
| *NR1I2* | rs7643645 | A>G | 0.345 | 0.476 | 0.179 |  | 0.588 | 0.177 | 0.235 |
| *PPARD* | rs2076169 | T>C | 0.793 | 0.193 | 0.014 |  | 0.686 | 0.257 | 0.057 |
| *PPARG* | rs2972164 | T>C | 0.246 | 0.531 | 0.223 |  | 0.412 | 0.412 | 0.176 |
| *PPARG* | rs880663 | T>C | 0.585 | 0.396 | 0.019 |  | 0.800 | 0.200 | 0.000 |
| *NFKB1* | rs230539 | A>G | 0.521 | 0.379 | 0.100 |  | 0.343 | 0.457 | 0.200 |
| *TNF* | rs3093662 | A>G | 0.848 | 0.138 | 0.014 |  | 0.686 | 0.314 | 0.000 |
| *VDR* | rs11168287 | A>G | 0.260 | 0.514 | 0.226 |  | 0.114 | 0.714 | 0.172 |
| *VDR* | rs11574026 | C>T | 0.802 | 0.179 | 0.019 |  | 0.657 | 0.343 | 0.000 |
| *VDR* | rs12717991 | G>A | 0.393 | 0.422 | 0.185 |  | 0.600 | 0.171 | 0.229 |

Abbreviations: mCRC, metastatic colorectal cancer; SNP, polymorphism; AA: homozygous for the more frequent allele; Aa: heterozygous, aa: homozygous for the variant allele.
