# Supplementary material for: Association of STAT-3 rs1053004 and VDR rs11574077 With FOLFIRI-Related Gastrointestinal Toxicity in Metastatic Colorectal Cancer Patients
Source: Front Pharmacol. 2018 Apr 13;9:367. doi: 10.3389/fphar.2018.00367 (PMC5908896; doi:10.3389/fphar.2018.00367)
Supplement: Supplementary file 3 [file Table_3.doc]

Supplementary Material

Title: *STAT-3* rs1053004 and *VDR* rs11574077 as predictors of gastrointestinal toxicity in metastatic colorectal cancer patients receiving first-line FOLFIRI treatment.

**Authors:** Elena De Mattia^1§^, Erika Cecchin^1§^, Marcella Montico^1^, Adrien Labriet^2^, Chantal Guillemette^2.3^, Eva Dreussi^1^, Rossana Roncato^1^, Alessia Bignucolo^1^, Angela Buonadonna^4^, Mario D’Andrea^5^, Luigi Coppola^6^, Sara  Lonardi^7^, Eric Lévesque^8^, Derek Jonker^9^, Félix Couture^8^, Giuseppe Toffoli^1*^

**Correspondence to:**

***Dr. Giuseppe Toffoli MD, Director,** Clinical and Experimental Pharmacology, CRO- National Cancer Institute, Via Franco Gallini n. 2, 33081 Aviano (PN) –Italy. [gtoffoli@cro.it](mailto:gtoffoli@cro.it)

Telephone +39-0434-659612 and Fax +39-0434-659799

**Supplementary Table S3**. **Genotype frequency distribution for grade 3-4 vs. grade 0-2 cumulative gastrointestinal toxicity in the discovery set (n=247 mCRC patients) according to gene polymorphisms.**

| **Genes** | **SNP** | **Base change** | **Genotype frequency** | | | | | | |
| --- | --- | --- | --- | --- | --- | --- | --- | --- | --- |
|  |  |  | **grade 0-2 (n=221)** | | |  | **grade 3-4 (n=26)** | | |
|  |  |  | **AA** | **Aa** | **aa** |  | **AA** | **Aa** | **aa** |
| *HNF4A* | rs1800961 | C>T | 0.986 | 0.014 | 0.000 |  | 0.885 | 0.115 | 0.000 |
| *HNF4A* | rs2071197 | G>A | 0.869 | 0.131 | 0.000 |  | 0.654 | 0.308 | 0.038 |
| *HNF4A* | rs6031587 | C>T | 0.896 | 0.104 | 0.000 |  | 0.731 | 0.192 | 0.077 |
| *HNF4A* | rs6093976 | C>T | 0.651 | 0.317 | 0.032 |  | 0.808 | 0.192 | 0.000 |
| *NR1I3* | rs2307424 | C>T | 0.471 | 0.407 | 0.122 |  | 0.192 | 0.731 | 0.077 |
| *NR1I3* | rs4073054 | T>G | 0.388 | 0.416 | 0.196 |  | 0.423 | 0.539 | 0.038 |
| *PPARA* | rs9626736 | A>G | 0.416 | 0.425 | 0.159 |  | 0.154 | 0.654 | 0.192 |
| *PPARD* | rs2076169 | T>C | 0.783 | 0.203 | 0.014 |  | 0.731 | 0.192 | 0.077 |
| *RXRG* | rs380518 | T>C | 0.832 | 0.145 | 0.023 |  | 0.615 | 0.385 | 0.000 |
| *RXRG* | rs4657437 | C>A | 0.394 | 0.468 | 0.138 |  | 0.500 | 0.462 | 0.038 |
| *RXRG* | rs283695 | G>A | 0.389 | 0.475 | 0.136 |  | 0.231 | 0.577 | 0.192 |
| *RXRG* | rs3767344 | G>C | 0.249 | 0.498 | 0.253 |  | 0.385 | 0.385 | 0.230 |
| *RXRG* | rs157880 | C>T | 0.751 | 0.235 | 0.014 |  | 0.769 | 0.154 | 0.077 |
| *RXRB* | rs2744537 | G>T | 0.670 | 0.298 | 0.032 |  | 0.654 | 0.231 | 0.115 |
| *VDR* | rs11574077 | A>G | 0.932 | 0.068 | 0.000 |  | 0.846 | 0.115 | 0.039 |
| *VDR* | rs4760648 | C>T | 0.356 | 0.480 | 0.164 |  | 0.154 | 0.577 | 0.269 |
| *VDR* | rs2853564 | T>C | 0.332 | 0.514 | 0.154 |  | 0.539 | 0.423 | 0.038 |
| *TNF* | rs1800629 | A>G | 0.227 | 0.773 | 0.000 |  | 0.080 | 0.920 | 0.000 |
| *TNF* | rs1800630 | C>A | 0.654 | 0.223 | 0.123 |  | 0.461 | 0.308 | 0.231 |
| *STAT3* | rs1053004 | T>C | 0.441 | 0.405 | 0.154 |  | 0.577 | 0.385 | 0.038 |
| *INFG* | rs2069716 | A>G | 0.808 | 0.192 | 0.000 |  | 0.960 | 0.040 | 0.000 |
| *IL6* | rs2069840 | C>G | 0.430 | 0.448 | 0.122 |  | 0.615 | 0.308 | 0.077 |

Abbreviations: mCRC, metastatic colorectal cancer; SNP, polymorphism; AA: homozygous for the more frequent allele; Aa: heterozygous, aa: homozygous for the variant allele.
