# Supplementary material for: Association of STAT-3 rs1053004 and VDR rs11574077 With FOLFIRI-Related Gastrointestinal Toxicity in Metastatic Colorectal Cancer Patients
Source: Front Pharmacol. 2018 Apr 13;9:367. doi: 10.3389/fphar.2018.00367 (PMC5908896; doi:10.3389/fphar.2018.00367)
Supplement: Supplementary file 4 [file Table_4.doc]

Supplementary Material

Title: *STAT-3* rs1053004 and *VDR* rs11574077 as predictors of gastrointestinal toxicity in metastatic colorectal cancer patients receiving first-line FOLFIRI treatment.

**Authors:** Elena De Mattia^1§^, Erika Cecchin^1§^, Marcella Montico^1^, Adrien Labriet^2^, Chantal Guillemette^2.3^, Eva Dreussi^1^, Rossana Roncato^1^, Alessia Bignucolo^1^, Angela Buonadonna^4^, Mario D’Andrea^5^, Luigi Coppola^6^, Sara  Lonardi^7^, Eric Lévesque^8^, Derek Jonker^9^, Félix Couture^8^, Giuseppe Toffoli^1*^

**Correspondence to:**

***Dr. Giuseppe Toffoli MD, Director,** Clinical and Experimental Pharmacology, CRO- National Cancer Institute, Via Franco Gallini n. 2, 33081 Aviano (PN) –Italy. [gtoffoli@cro.it](mailto:gtoffoli@cro.it)

Telephone +39-0434-659612 and Fax +39-0434-659799

**Table S4.** **Association of selected polymorphisms with pharmacokinetic (PK) parameters in a discovery cohort**  **subset population (N=71).** Only the association with a *p*-value <0.1 are reported.

| **Genes** | **SNP** | **Base**  **change** | **Model** | **PK parameter** | **Effect** | **AUC mean values** | | | **Mean values according to the model** | ***p*-value** | **Effect on toxicity** |
| --- | --- | --- | --- | --- | --- | --- | --- | --- | --- | --- | --- |
|  |  |  |  |  |  | **AA** | **Aa** | **aa** |  |  |  |
| *HNF4A* | rs6031587 | C>T | Dominant** | CPT-11 AUC | Increased | 18.67 | 27.00 | 22.73 | AA: 18.67 vs. Aa+aa: 26.29 | 0.091 | Increased GI (additive model) |
| *RXRG* | rs3767344 | G>C | Dominant | GR | Increased | 3.09 | 4.38 | 5.22 | AA: 3.09 vs. Aa+aa: 4.67 | 0.078 | Inferior GI (dominant model) |
| *VDR* | rs12717991 | G>A | Dominant | GR | Increased | 3.94 | 5.10 | 4.10 | AA: 3.94 vs. Aa+aa: 4.7 | 0.073 | Inferior neutropenia (dominant model) |
| *VDR* | rs11574077 | A>G | Additive | BI | Increased | 5.71 | 9.65 | // | AA: 5.71 vs. Aa: 9.65 | 0.036 | Increased GI (additive model) |
|  |  |  |  | GR | Inferior | 4.48 | 1.97 | // | AA: 4.48 vs. Aa: 1.97 | 0.012 |  |

** Additive model was not applied due to the low number (n=2) of homozygous polymorphic individuals.

**Abbreviations:** AUC, area under the curve; BI, biliary index [CPT-11 AUC X (SN38 AUC/SN38G AUC)]; CPT-11: irinotecan; GI, gastrointestinal toxicity; GR: glucuronidation ratio (SN-38G AUC/SN-38 AUC); SN-38: active form of irinotecan; SN-38G: inactive glucuronidation form of irinotecan.
